# Association of the Composite Dietary Antioxidant Index and Consumption Time with NAFLD: The U.S. National Health and Nutrition Examination Survey, 2017–2020

**DOI:** 10.3390/nu16203556

**Published:** 2024-10-20

**Authors:** Kening Zhang, Yan Xu, Nan Zhang, Xi Liang, Huaqi Zhang, Hui Liang

**Affiliations:** Department of Nutrition and Food Hygiene, School of Public Health, Qingdao University, 308 Ningxia Road, Qingdao 266071, China; 2024010107@qdu.edu.cn (K.Z.); 2021010149@qdu.edu.cn (Y.X.); 2023010076@qdu.edu.cn (N.Z.); liangxi@qdu.edu.cn (X.L.); huaqi_erin_qy@qdu.edu.cn (H.Z.)

**Keywords:** meal timing, NHANES, NAFLD, CDAI

## Abstract

Background: The timing of food intake can affect the physiological and metabolic functions of the body. However, whether and how the timing of dietary antioxidant intake could influence non-alcoholic fatty liver disease (NAFLD) is largely unknown. The Composite Dietary Antioxidant Index (CDAI) serves as a comprehensive measure that encompasses various dietary antioxidants. This study aims to investigate the association between the meal timing of CDAI and NAFLD in American adults. Methods: We used data from the 2017–2020 National Health and Nutrition Examination Survey (NHANES). Dietary intake was assessed through the implementation of two non-concurrent 24-h dietary recalls. Vibration-controlled transient elastography was employed to assess the controlled attenuation as an indicator of NAFLD. CDAI across the day (total, breakfast, lunch, dinner) and Δ CDAI (Δ = dinner-breakfast) were categorized into quartiles. Weighted logistic regression models and restricted cubic splines were used to evaluate the association between the meal timing of CDAI and NAFLD. Results: Of the 6570 participants in this study, 1153 had NAFLD. Participants in the highest quartile of total CDAI levels had a lower risk of NAFLD compared with the lowest quartile (OR = 0.52; 95% CI, 0.38–0.71). More importantly, participants in the highest quartile of dinner CDAI, but not those in that of breakfast or lunch, had a lower risk of NAFLD (OR = 0.54; 95% CI, 0.40–0.73) compared with the lowest quartile. The restricted cubic splines indicated a linear relationship between total CDAI and NAFLD (*P*_for nonlinearity_ = 0.70), as well as between dinner CDAI and NAFLD (*P*_for nonlinearity_ = 0.19). Stratification analyses revealed that the effect of dinner CDAI on NAFLD varied between non-Hispanic Whites and individuals of other races (*P*_for interaction_ = 0.032). Conclusions: these findings suggest the potential beneficial effects of an antioxidant-rich diet and strategic meal timing on NAFLD.

## 1. Introduction

Non-alcoholic fatty liver disease (NAFLD) has been observed to affect about 25% of the global population over the past four decades, and it is closely associated with metabolic dysfunctions, including obesity, hyperlipidemia, diabetes, and hypertension [1]. Due to its extensive metabolic implications, NAFLD should be considered a significant public health concern [2]. The current therapeutic strategies for NAFLD primarily focus on the mitigation of the disease by addressing associated risk factors, such as obesity, dyslipidemia, inflammation, and oxidative stress [3]. Previous studies have reported that oxidative stress and mitochondrial damage can contribute to hepatic dysfunction, as well as NAFLD initiation and progression [4,5,6].

At present, the “multiple-hit” hypothesis, an updated theoretical framework that incorporates various concurrently acting factors, offers a more comprehensive explanation for the pathogenesis of NAFLD. Among the numerous factors contributing to the “multiple hits” hypothesis, oxidative stress is regarded as the primary contributor to liver injury and the progression of NAFLD [7,8].The generation of reactive oxygen species (ROS) is of critical importance in various physiological processes, as it regulates cellular homeostasis across the spectrum from health to disease [9].The accumulation of fat induces metabolic disturbances, leading to increased mitochondrial ROS production and endoplasmic reticulum stress, which subsequently can result in inflammation, cellular injury, and apoptosis [10]. This mechanism is highlighted by the observation that patients with NAFLD frequently exhibit s compromised antioxidant status, characterized by reduced serum concentrations of the antioxidants vitamin E (tocopherol) and vitamin C, alongside elevated levels of lipid peroxidation products and systemic oxidative stress markers [11,12]. Previous studies have suggested that dietary antioxidants can reduce oxidative stress and histologically improve liver function [13,14]. Some micronutrients, including zinc, iron, selenium, magnesium, and vitamins A, C, D, and E, as well as carotenoids, have beneficial effects on NAFLD through their lipo-protective, antioxidant, antifibrotic, and immunomodulatory properties [4]. Several observational studies have examined the associations between dietary antioxidant nutrients and NAFLD, such as vitamins A, C, E and carotenoids [15,16,17]. However, some conflicting reports have shown that a single dietary antioxidant nutrient may not be associated, and could even have adverse effects [15,18]. A previous study showed that oral supplementation with vitamin A could provoke oxidative stress, disrupt redox homeostasis, impair antioxidant capacity, and induce inflammation in rats, contrary to its traditionally recognized role as a protective antioxidant [19]. The inconsistent findings suggest that isolated compounds may not be effective in mitigating diseases associated with oxidative stress [20]. The Composite Dietary Antioxidant Index (CDAI), as proposed by Wright et al. [21], serves as a comprehensive measure that encompasses various dietary antioxidants and reflects an individual’s overall dietary antioxidant intake profile. The development of CDAI was based on its collective impact on anti-inflammatory responses, specifically targeting pro-inflammatory markers such as Tumor Necrosis Factor-α (TNF-α) and Interleukin-1β (IL-1β). These markers have been associated with numerous health outcomes, including hypertension, depression, and all-cause mortality [22,23,24].

In recent years, emerging evidence has suggested that meal timing can affect the physiological and metabolic functions of the body [25], possibly due to the role of the circadian rhythm. The circadian timing system is regulated by a primary clock located in the suprachiasmatic nucleus (SCN) within the hypothalamus, which interacts through neuroendocrine pathways with various peripheral clocks, such as those found in the liver. Although the primary pacemaker located in the SCN is primarily regulated by the light-dark cycle, the circadian clock within the liver exhibits sensitivity to dietary patterns [26]. In fact, the circadian phase of hepatic function can be directly affected by feeding habits, and this modulation occurs independently of both the SCN and light–dark signaling pathways [27,28]. Modifying dietary composition and eating patterns may potentially serve as a viable strategy for the treatment of NAFLD by mitigating oxidative stress levels in the body. Several studies have indicated that the distribution of vitamin, macronutrient, and mineral intake throughout the day plays a significant role in the regulation of the metabolic state and body weight [29,30,31]. The close interplay between the circadian clock and metabolism underscores the significance of meal timing as a determinant of metabolic regulation [32].

Based on these findings, we hypothesized that the meal timing of CDAI may be closely associated with NAFLD. This study aimed to evaluate the association between CDAI across the day (total, breakfast, lunch, dinner) and Δ CDAI (Δ = dinner–breakfast) and NAFLD among the participants of the U.S. National Health and Nutrition Examination Survey (NHANES).

## 2. Methods

### 2.1. Data Source and Study Population

The NHANES is a comprehensive, multistage, stratified, and clustered probability survey that utilizes a sample of the noninstitutionalized civilian population in the United States, which is representative of the entire nation. Information on vibration-controlled transient elastography (VCTE) was provided only in the two cycles of NHANES 2017–2020, and thus it was extracted as identified data. The NHANES has received approval from the Institutional Review Board of the Centers for Disease Control and Prevention, ensuring compliance with ethical guidelines. All data and documentation related to the study are accessible to the public. Furthermore, participants in the study have provided their informed consent by signing consent forms.

**Figure 1:**
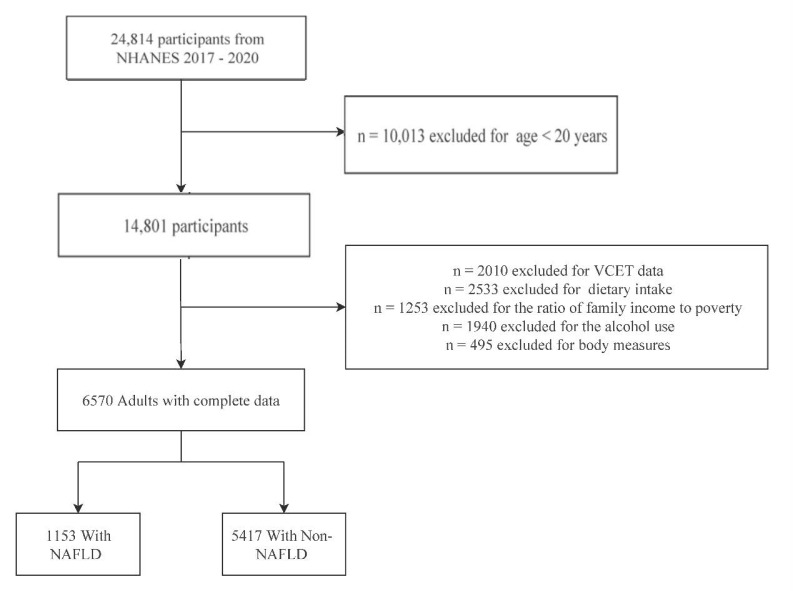
Flow diagram of study cohort selection from NHANES: 2017–2020.

A total of 24,814 individuals were sampled in the NHANES 2017–2018 and 2019–2020 datasets. In this study, the following criteria were applied for exclusion: (i) participants under the age of 20; (ii) missing data for self-reported personal interviews; (iii) missing data for VCTE; and (iv) missing data for dietary intake. Ultimately, 6570 individuals remained and were included as participants in the current study. A flowchart of participant enrollment is shown in Figure 1.

### 2.2. Assessment of Dietary Intake

Dietary intake was assessed using two non-concurrent 24-h dietary recalls. The initial recall was administered face-to-face, while the subsequent recall was completed via telephone within a period of 3 to 10 days. Estimation of dietary nutrients and energy intake was accomplished by utilizing the Food and Nutrient Database for Dietary Studies, provided by the U.S. Department of Agriculture. The consumption of absolute foods and macronutrients was adjusted for total energy intake using the residual method to correct measurement errors in dietary estimates [33]. The variable “Name of eating occasion” divided the foods and macronutrients into breakfast, lunch, and dinner according to the time of intake of the foods and macronutrients. Decisions regarding the classification of eating events were determined by the event label employed by the respondent to characterize said events. This approach offers comprehensive definitions that take into account diverse social norms and cultural practices [30]. The average dietary consumption from 2017 to 2020 during the 2 days was calculated.

### 2.3. Composite Dietary Antioxidant Index Calculation

The exposure variables in this study included the meal timing of CDAI (total, breakfast, lunch, and dinner) and the difference between dinner CDAI and breakfast CDAI (Δ = dinner − breakfast). In this study, six dietary antioxidants were included in the analysis: carotenoids, selenium, zinc, and vitamins A, C, and E. Following the measurement method proposed by Wright, we used a modified version of the CDAI [21,34,35]. Briefly, normalization for each of the six dietary antioxidants involved subtracting the mean intake from each antioxidant and dividing by the standard deviation. Subsequently, we summed up the standardized dietary antioxidant intake, and the conversion did not incorporate dietary antioxidants intake from medications and dietary supplements.

$$\mathrm{CDAI}=\sum_{i=1}^{6}\;\frac{x_{i}-\mu_{i}}{S_{i}}$$

In this formula, *x_i_* represents the daily consumption of antioxidants, *μ_i_* denotes the average level of antioxidants within the study cohort, and *S_i_* signifies the standard deviation of *μ_i_*.

### 2.4. Definition of NAFLD

VCTE is used to quantify ultrasound attenuation associated with hepatic steatosis and to measure the controlled attenuation parameter (CAP) as an indicator of hepatic fat content. The accuracy of CAP for evaluating liver steatosis and fibrosis has been assessed by previous researchers. In this study, NAFLD was defined as a CAP value of ≥285 dB/m, as this threshold has been shown to have 80% sensitivity and 77% specificity in accurately identifying any level of liver steatosis [36].

### 2.5. Covariates

Based on the previous literature, the following potential covariates were included: age, sex, race/ethnicity, educational level, poverty to income ratio (PIR), marital status, body mass index (BMI), waist circumference, smoking status, drinking status, physical activity, systolic pressure (SBP), diastolic pressure (DBP), total energy intake, dietary supplement use, and diabetes mellitus. As used by NHANES, race/ethnicity was classified into four categories: non-Hispanic White, non-Hispanic Black, Mexican American, and other races. Education level was categorized as less than high school, high school or equivalent, and college or more. PIR was stratified into three groups: less than 1.3, 1.3 to 3.5, and equal to or greater than 3.5. Marital status was categorized into three groups: never married, widowed/divorced/separated, and married/cohabiting. Smoking status was defined as either not currently smoking or currently smoking. Participants were categorized into three groups: nondrinkers, low to moderate drinkers, and heavy drinkers, according to their self-reported average daily alcohol consumption. Low to moderate drinkers were defined as an individual consuming less than 2 drinks per day for men and less than 1 drink per day for women; conversely, heavy drinkers were defined as an individual consuming 2 or more drinks per day for men and 1 or more drinks per day for women. Physically active participants were defined as engaging in moderate-intensity or vigorous sports, fitness programs, or recreational activities for a duration exceeding 10 min per week. Conversely, individuals who failed to meet this threshold were classified as inactive [37]. Dietary supplement use was defined as a response to the question, “Any dietary supplements taken?”. Diabetes was ascertained based on the utilization of diabetes medication or insulin, self-reported diabetes history, fasting glucose levels exceeding 7 mmol/L or glycohemoglobin A1c levels surpassing 6.5%, random blood glucose levels equal to or exceeding 11.1 mmol/L, or two-hour OGTT (Oral Glucose Tolerance Test) blood glucose levels equal to or exceeding 11.1 mmol/L. Detailed measurement procedures and standards can be found at https://www.cdc.gov/nchs/nhanes, accessed on 8 October 2023.

### 2.6. Statistical Analysis

According to the NHANES analytic guidelines, all analyses incorporated sample weights, stratification, and clustering to account for the other aspects of the complex survey design. Total CDAI and dinner CDAI were categorized into quartiles for baseline description, and the lowest quartile was set as the reference group. Demographic characteristics, dietary nutrient intake, and physical measurements were presented as mean (95% CI) for continuous variables and percentage (95% CI) for categorical variables. The chi-square test was used to compare CDAI quartiles for categorical variables, while ANOVA was employed for continuous variables. Logistic regression models were performed to evaluate the relationship between CDAI (total, breakfast, lunch, dinner, Δ CDAI) and NAFLD. Model 1 was a crude model, and model 2 was adjusted for demographic characteristics, including age, sex, race/ethnicity, education level, PIR, and marital status. Model 3 was further adjusted for BMI, smoking status, alcohol use, waist circumference, SBP, DBP, total energy intake, dietary supplement use, physical activity, and diabetes. The restricted cubic spline analysis (RCS) with three knots (at the 10th, 50th, and 90th percentiles) was performed to investigate the nonlinear associations between the CDAI and NAFLD. Moreover, the study conducted stratification and interaction analyses to investigate potential variations in these associations across different age groups, race/ethnicity, education levels, PIR, smoking status, and obesity status (obesity, BMI < 30 kg/m^2^; or non-obesity, BMI ≥ 30 kg/m^2^) in the fully adjusted model. All statistical analyses were performed using R 4.2.2, and a two-sided *p*-value less than 0.05 was deemed statistically significant.

## 3. Results

### 3.1. Characteristics of the Participants

The study sample comprised 6570 individuals, with a mean age of 47.09 years. The participants had an average CADI score of 0.18, ranging from −7.668 to 53.573. The baseline characteristics of participants are presented in Table 1, categorized according to quartiles of dinner CDAI. Participants in the highest dinner CDAI quartile were more likely to be older, non-smokers, men, and use dietary supplements, with higher total energy intake as well as lower alcohol intake, in comparison to quartiles 1–3. Moreover, as dinner CDAI levels increased, the prevalence of NAFLD decreased. Additionally, significant differences in baseline characteristics were observed in terms of race/ethnicity, education level, PIR, marital status, and dietary antioxidants. The baseline characteristics of total CDAI by quartiles are presented in Supplementary Table S1.

### 3.2. The Association between Composite Dietary Antioxidant Index and NAFLD

Table 2 presents the results of the logistic regression weighted model, which examined the relationship between CDAI (total, breakfast, lunch, dinner, Δ CDAI) and NAFLD. In the fully adjusted model, participants in the highest quartile of total CDAI level had a lower risk of NAFLD compared with the lowest quartile (OR = 0.52; 95% CI, 0.38–0.71) (*P*_for trend_ < 0.001). Compared to the lowest quartile of dinner CDAI, participants in the highest quartile had a lower risk of NAFLD (OR = 0.54; 95% CI, 0.40–0.73) (*P*_for trend_ < 0.001). However, this significant association did not occur between CDAI from breakfast (OR = 0.88; 95% CI, 0.64–1.20) (*P*_for trend_ = 0.180) or lunch (OR = 0.82; 95% CI, 0.57–1.18) (*P*_for trend_ = 0.291) and NAFLD. The association of Δ CDAI with NAFLD was nonsignificant but displayed a trend. (OR = 0.83; 95% CI, 0.66–1.05) (*P*_for trend_ = 0.045).

**Figure 2:**
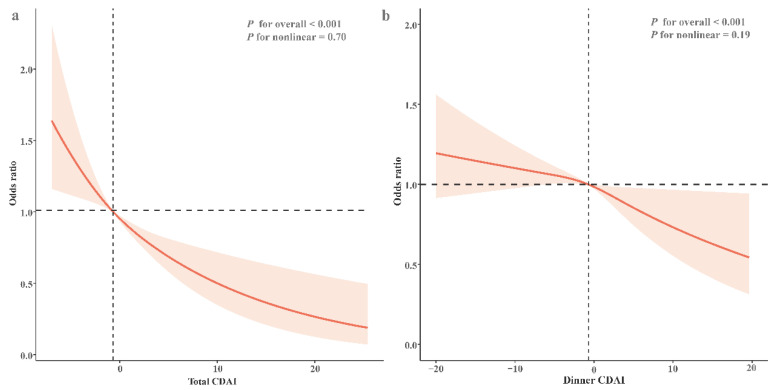
(**a**) The restricted cubic regression between total CDAI and NAFLD. (**b**) The restricted cubic regression between dinner CDAI and NAFLD.

Furthermore, RCS curve results indicated a linear association between total CDAI level and NAFLD (*P*_for nonlinearity_ = 0.70; Figure 2a), as well as between dinner CDAI level and NAFLD (*P*_for nonlinearity_ = 0.19; Figure 2b), in the fully adjusted model.

### 3.3. Subgroup Analysis

The results of subgroup analyses to assess the association between total CDAI level and NAFLD were presented in Table 3. This inverse association persisted in the subgroup analysis based on age group, sex, and race/ethnicity, as well as in participants not currently smoking, with non-obesity and a PIR greater than or equal to 1.8. However, a significant interaction between total CDAI level and education level for NAFLD was observed (*P*_interaction_ = 0.002). As shown in Table 4, higher dinner CDAI was related to a lower NAFLD risk in participants of other races rather than Non-Hispanic White (*P*_interaction_ = 0.032). Additionally, no significant interaction was observed between dinner CDAI and any of the stratifying variables.

## 4. Discussion

In this nationwide cross-sectional survey based on NHANES, we found inverse correlations between total CDAI, dinner CDAI, and NAFLD, adjusting for sociodemographic characteristics, physical measurements, dietary factors, and comorbidity covariates. The restricted cubic spline showed that the relationship between total CDAI and NAFLD, as well as between dinner CDAI and NAFLD, was linear.

To our knowledge, this is the first study to explore the correlation between the meal timing of CDAI and NAFLD, utilizing nationally representative data. Furthermore, it underscores the significance of CDAI distribution in mitigating the risk of NAFLD. Although the current research on the correlation between CDAI and NAFLD remains limited, there has been extensive discussion on the advantageous impact of dietary antioxidants. A case-control study revealed an inverse relationship between dietary total antioxidant capacity (TAC) and NAFLD [38]. This observation is consistent with our research results. In a cross-sectional study conducted on patients with NASH, those with the highest dietary TAC exhibited a decrease of approximately 20% in the risk of having many ballooned hepatocytes [39]. It is recommended that the consumption of micronutrients, which possess anti-oxidative and anti-inflammatory properties, be increased for the prevention and treatment of NAFLD [40,41]. As common antioxidants, vitamin E and vitamin C are widely recognized and frequently employed interventions in patients with NAFLD, and previous studies have reported that the beneficial effects of vitamin E/C combinations in NAFLD/NASH [42,43]. The precise mechanisms underlying the protective effects of carotenoids in NAFLD remain uncertain. However, a multitude of experimental studies have suggested that carotenoids may exert their effects through various pathways, encompassing antioxidant and anti-inflammatory actions [44,45]. In fact, several animal studies have shown that carotenoids, including β-carotene, lycopene, lutein, and β-cryptoxanthin, exhibit antioxidant effects against lipid peroxidation within the hepatic system [46,47,48,49]. Meanwhile, a previous animal study reported the potential of dietary selenium to mitigate liver damage and insulin resistance during the progression of NAFLD, as well as its ability to alleviate oxidative stress in hepatocytes [50].

In addition, this study further demonstrated that CDAI level at dinner rather than breakfast or lunch was inversely related to NAFLD. Some biological processes may partly explain this association. For example, genes associated with oxidative stress and inflammation exhibit circadian rhythms, potentially influencing the hepatic antioxidant and inflammatory state [51,52]. The intake of dinner CDAI may play a role in mitigating oxidative stress and the inflammatory response by aligning with circadian rhythms, which in turn could enhance health benefits. Several prominent antioxidants (GSH-PX, MDA, SOD, and melatonin) and white blood cell types (lymphocytes, neutrophils, and monocytes) in humans have been documented to exhibit peak levels during the nocturnal period or prior to daybreak [53]. The temporal shifts in inflammation peaks align with the timing of CDAI intake during the evening, probably contributing to the reduction in NAFLD risk. Previous studies have shown that an augmented intake of vitamins C and E during dinner may potentially reduce the risk of NAFLD, plausibly due to the greater compatibility of higher vitamins C and E consumption during dinner with the circadian rhythm of antioxidant activity [31]. Meanwhile, vitamin A has been demonstrated to play a role in regulating the circadian patterns of various antioxidant enzymes, such as catalase (CAT), glutathione peroxidase (GPx), and glutathione reductase (GR) [54,55].

Subgroup analysis revealed that individuals with higher levels of education exhibited a more pronounced protective effect of total CDAI, potentially due to their inclination towards adopting healthier lifestyles. Individuals from other races were found to obtain health benefits as a result of consuming dinner CDAI, which could be attributed to the fact that home dinner preparation habits varied substantially with various social norms and cultural behaviors. For instance, consumption of traditional rice-based dinners has been linked to a heightened risk of hyperglycemia [56]. A cross-sectional study showed that the habits associated with home dinner preparation exhibit significant variation according to socio-economic status and race/ethnicity [57]. These associations are likely to have important implications for the design and appropriate tailoring of interventions aimed at enhancing home food preparation practices and promoting healthy eating.

This study has several strengths. First, this is the first study investigating the association between the meal timing of CDAI and NAFLD. Furthermore, the CDAI was implemented in order to assess the comprehensive antioxidant capacity of the diet, taking into account the combined impact of various antioxidant substances present in diverse food items.

However, this study also has several limitations. First, as this is a cross-sectional study, we could not establish causal inferences. Furthermore, self-reported dietary 24-h recall data per person may be limited in characterizing diet over a person’s lifespan and are subject to measurement errors due to large day-to-day variations in food intake. Finally, we used diagnostic criteria for NAFLD, rather than the more commonly used metabolic dysfunction-related fatty degenerative liver disease.

## 5. Conclusions

The findings of this cross-sectional study suggested total CDAI and dinner CDAI were inversely associated with NAFLD risk in American adults after adjustment for potential confounding factors. Our findings suggest the potential beneficial effects of an antioxidant-rich diet and strategic meal timing on NAFLD. The optimal intake time of dietary antioxidants was at dinner. In the future, large-scale prospective studies are required to validate these findings and enhance the precision and efficacy of prevention and treatment strategies for NAFLD.

## Figures and Tables

**Table 1:** Baseline characteristics stratified by quartile of dinner CDAI (*n* = 6570) in the NHANES.

	Quartile 1 (N = 1643)	Quartile 2 (N = 1648)	Quartile 3 (N = 1637)	Quartile 4 (N = 1642)	*p*-Value
Age, years	46.55 (45.12, 47.97)	45.47 (43.76, 47.18)	47.44 (46.44, 48.44)	48.71 (47.26, 50.16)	0.001
Male, %	37.98 (34.40, 41.55)	44.36 (39.87, 48.85)	44.83 (41.70, 47.96)	62.16 (59.31, 65.01)	<0.001
Race/ethnicity, %					<0.001
Non-Hispanic White	57.01 (52.35, 61.67)	64.43 (60.24, 68.62)	69.58 (64.95, 74.22)	66.98 (62.99, 70.96)	
Non-Hispanic Black	15.35 (12.67, 18.02)	11.21 (8.45, 13.97)	9.67 (7.13, 12.21)	9.04 (6.75, 11.32)	
Mexican American	8.25 (6.10, 10.40)	8.59 (6.27, 10.91)	6.76 (4.91, 8.61)	6.50 (4.78, 8.23)	
Other races	19.40 (16.55, 22.25)	15.77 (13.21, 18.32)	13.99 (11.29, 16.70)	17.48 (14.46, 20.50)	
Smoking status, %					0.05
Non-smoker	80.98 (77.25, 84.70)	84.57 (82.26, 86.87)	84.87 (81.84, 87.89)	85.68 (83.18, 88.17)	
Current smoker	19.02 (15.30, 22.75)	15.43 (13.13, 17.74)	15.13 (12.11, 18.16)	14.32 (11.83, 16.82)	
Poverty to income ratio, %					<0.001
<1.3	21.97 (19.24, 24.70)	18.89 (16.37, 21.41)	15.03 (13.05, 17.02)	14.59 (11.86, 17.32)	
1.3–3.49	36.76 (32.99, 40.53)	31.52 (27.83, 35.22)	32.11 (27.22, 36.99)	33.15 (28.33, 37.97)	
≥3.5	41.27 (36.73, 45.80)	49.59 (45.47, 53.70)	52.86 (47.32, 58.39)	52.26 (47.55, 56.97)	
Alcohol status, %					0.1
None	10.66 (8.82, 12.51)	8.55 (6.43, 10.67)	6.40 (4.70, 8.09)	6.30 (3.76, 8.84)	
Low to moderate	64.92 (60.79, 69.06)	68.01 (63.07, 72.96)	70.70 (66.58, 74.82)	72.39 (68.95, 75.83)	
Heavy	24.41 (20.49, 28.34)	23.44 (19.65, 27.23)	22.90 (18.87, 26.94)	21.31 (18.02, 24.61)	
Educational level, %					0.002
Less than high school	10.33 (8.37, 12.30)	8.36 (6.41, 10.32)	7.04 (5.71, 8.38)	5.57 (4.13, 7.01)	
High school or equivalent	31.89 (27.25, 36.53)	26.75 (22.94, 30.55)	22.36 (19.39, 25.33)	25.76 (22.01, 29.50)	
College or more	57.78 (52.87, 62.68)	64.89 (60.10, 69.68)	70.60 (67.21, 73.98)	68.67 (63.98, 73.36)	
Marital status, %					<0.001
Never married	23.94 (20.81, 27.08)	20.75 (16.65, 24.86)	14.56 (11.95, 17.17)	17.92 (14.48, 21.35)	
Widowed/divorced/separated	18.97 (16.08, 21.85)	21.97 (20.02, 23.92)	15.26 (13.38, 17.13)	14.76 (12.14, 17.38)	
Married/cohabiting	57.09 (53.27, 60.91)	57.28 (52.99, 61.57)	70.19 (67.19, 73.18)	67.32 (62.67, 71.98)	
Physical activity, %					0.05
Inactive	53.69 (50.67, 56.71)	52.18 (47.69, 56.67)	45.71 (42.59, 48.83)	50.14 (45.35, 54.94)	
Active	46.31 (43.29, 49.33)	47.82 (43.33, 52.31)	54.29 (51.17, 57.41)	49.86 (45.06, 54.65)	
NAFLD, %	20.02 (16.86, 23.19)	16.13 (12.73, 19.53)	13.51 (11.11, 15.92)	12.87 (10.40, 15.34)	0.01
Diabetes, %	14.03 (11.03, 17.03)	13.63 (11.22, 16.05)	12.48 (10.51, 14.44)	14.12 (11.13, 17.10)	0.80
Body mass index, kg/m^2^	29.73 (29.10, 30.36)	30.08 (29.51, 30.66)	29.80 (29.38, 30.22)	29.58 (28.79, 30.37)	0.74
Total energy intake, kcal/day	1621.53 (1575.03, 1668.04)	2028.04 (1987.09, 2069.00)	2297.45 (2235.81, 2359.10)	2634.30 (2532.38, 2736.23)	<0.001
Waist circumference, cm	99.68 (98.30, 101.07)	101.03 (99.45, 102.61)	100.44 (99.23, 101.64)	101.39 (99.30, 103.47)	0.43
SBP, mmHg	121.62 (120.10, 123.15)	120.27 (118.85, 121.70)	121.14 (119.97, 122.31)	120.92 (119.81, 122.03)	0.57
DBP, mmHg	74.75 (73.89, 75.62)	74.30 (73.35, 75.24)	73.59 (72.77, 74.41)	73.84 (73.08, 74.60)	0.12
CDAI	−2.71 (−2.90, −2.52)	−1.15 (−1.36, −0.94)	0.35 (0.15, 0.55)	3.61 (3.34, 3.88)	<0.001
Vitamin A, µ g	413.71 (381.52, 445.89)	536.67 (498.58, 574.76)	637.38 (600.80, 673.96)	926.18 (858.48, 993.89)	<0.001
Vitamin C, mg	44.78 (41.05, 48.52)	56.39 (52.33, 60.45)	80.13 (75.17, 85.10)	111.21 (103.95, 118.46)	<0.001
Vitamin E, mg	6.35 (6.01, 6.69)	7.97 (7.58, 8.36)	9.93 (9.46, 10.39)	12.95 (12.27, 13.63)	<0.001
Zinc, mg	7.76 (7.39, 8.13)	10.14 (9.61, 10.67)	11.36 (11.03, 11.69)	14.28 (13.74, 14.82)	<0.001
Selenium, µ g	82.36 (79.50, 85.21)	107.69 (104.80, 110.57)	116.03 (112.74, 119.31)	149.63 (143.99, 155.27)	<0.001
Carotenoid, µ g	5853.50 (5065.27, 6641.72)	7109.78 (6436.59, 7782.98)	9246.94 (8679.75, 9814.13)	16,057.86 (14,440.98, 17,674.74)	<0.001
Dietary supplements use, %	48.27 (44.01, 52.52)	49.56 (45.30, 53.82)	62.87 (58.51, 67.23)	64.20 (60.15, 68.24)	0.001

Data are presented as mean (SD) or *n* (%). CDAI, the composite dietary antioxidant index; DBP, diastolic blood pressure; NAFLD, non-alcoholic fatty liver disease; SBP, systolic blood pressure.

**Table 2:** Associations of NAFLD with quartiles of meal timing of CDAI.

	Case/*n*	Model 1	Model2	Model 3
Total CDAI				
Q1	353/1643	Ref	Ref	Ref
Q2	332/1642	0.88 (0.72, 1.08)	0.87 (0.71, 1.07)	0.93 (0.74, 1.16)
Q3	255/1644	0.57 (0.41, 0.77) **	0.58 (0.43, 0.80) **	0.65 (0.45, 0.93) *
Q4	217/1641	0.53 (0.41, 0.69) ***	0.53 (0.40, 0.69) ***	0.52 (0.38, 0.71) **
*P*_for trend_				<0.001
Breakfast CDAI				
Q1	276/1643	Ref	Ref	Ref
Q2	316/1643	1.34 (1.04, 1.72)	1.15 (0.87, 1.52)	1.30 (0.96, 1.77)
Q3	295/1643	1.29 (1.04, 1.58)	1.06 (0.86, 1.32)	1.17 (0.90, 1.51)
Q4	266/1641	0.92 (0.70, 1.21)	0.82 (0.63, 1.05)	0.88 (0.64, 1.20)
*P*_for trend_				0.180
Lunch CDAI				
Q1	323/1643	Ref	Ref	Ref
Q2	306/1642	0.85 (0.66, 1.09)	0.85 (0.68, 1.06)	1.01 (0.80, 1.28)
Q3	299/1643	0.81 (0.59, 1.10)	0.86 (0.63, 1.17)	1.10 (0.78, 1.57)
Q4	225/1642	0.60 (0.45, 0.81) *	0.67 (0.49, 0.91) *	0.82 (0.57, 1.18)
*P*_for trend_				0.291
Dinner CDAI				
Q1	329/1644	Ref	Ref	Ref
Q2	297/1641	0.77 (0.53, 1.11)	0.82 (0.57, 1.18)	0.77 (0.53, 1.12)
Q3	295/1642	0.62 (0.46, 0.84) *	0.63 (0.45, 0.87) *	0.61 (0.43, 0.89) *
Q4	232/1643	0.59 (0.44, 0.79) **	0.59 (0.45, 0.79) **	0.54 (0.40, 0.73) **
*P*_for trend_				<0.001
Δ CDAI				
Q1	299/1643	Ref	Ref	Ref
Q2	286/1644	1.11 (0.85, 1.45)	1.08 (0.82, 1.44)	1.05 (0.76, 1.44)
Q3	324/1640	0.93 (0.75, 1.15)	0.98 (0.78, 1.23)	0.88 (0.70, 1.10)
Q4	244/1643	0.85 (0.67, 1.09)	0.89 (0.71, 1.10)	0.83 (0.66, 1.05)
*P*_for trend_				0.045

Model 1 was a crude model. Model 2 was adjusted for age, sex, race/ethnicity, education level, PIR and marital status. Model 3 was further adjusted for BMI, smoking status, alcohol use, waist circumference, SBP, DBP, total energy intake, dietary supplement use, physical activity and diabetes. * *p* < 0.05; ** *p* < 0.01; *** *p* < 0.001.

**Table 3:** Subgroup analyses of associations between total CDAI and NAFLD.

Subgroups	Quartile 1	Quartile 2	Quartile 3	Quartile 4	*P*_for interaction_
Age					0.078
20–59 years	Ref	1.05 (0.75, 1.48)	0.74 (0.46, 1.18)	0.56 (0.35, 0.92) *	
≥60 years	Ref	0.64 (0.37, 1.09)	0.49 (0.27, 0.86) *	0.47 (0.25, 0.87) *	
Sex					0.422
Female	Ref	1.14 (0.74, 1.75)	0.66 (0.38, 1.17)	0.53 (0.31, 0.90) *	
Male	Ref	0.65 (0.45, 0.93) *	0.38 (0.21, 0.66) *	0.44 (0.24, 0.78) *	
Race/ethnicity					0.367
Non-Hispanic White	Ref	0.73 (0.51, 1.03)	0.55 (0.33, 0.93) *	0.49 (0.30, 0.80) *	
Other races	Ref	0.99 (0.66, 1.48)	0.81 (0.58, 1.15)	0.50 (0.32, 0.78) *	
Education level					0.002
Less than high school	Ref	0.56 (0.27, 1.16)	0.19 (0.08, 0.49) *	0.41 (0.14, 1.21)	
High school or above	Ref	0.91 (0.72, 1.16)	0.68 (0.45, 1.05)	0.51 (0.36, 0.72) *	
Poverty to income ratio					0.798
<2.5	Ref	1.32 (0.86, 2.03)	0.78 (0.48, 1.27)	0.85 (0.57, 1.29)	
≥2.5	Ref	0.91 (0.64, 1.29)	0.34 (0.21, 0.58) *	0.35 (0.20, 0.61) *	
Smoking status					0.314
No	Ref	0.84 (0.67, 1.05)	0.54 (0.36, 0.83) *	0.37 (0.23, 0.60) *	
Yes	Ref	2.27 (1.03, 5.01) *	1.94 (0.97, 3.88)	1.00 (0.42, 2.41)	
Obesity status					0.836
Obesity	Ref	1.31 (0.84, 2.04)	0.68 (0.43, 1.08)	0.83 (0.55, 1.26)	
Non-obesity	Ref	0.69 (0.37, 1.27)	0.41 (0.26, 0.67) *	0.27 (0.13, 0.57) **	

Adjustments included age, sex, race/ethnicity, total energy intake, BMI, waist circumference, PIR, education, smoking status, alcohol use, exercise, marital status, systolic and diastolic blood pressure, dietary supplement use, and diabetes mellitus status by excluding the corresponding stratified variables. * *p* < 0.05; ** *p* < 0.01.

**Table 4:** Subgroup analyses of associations between dinner CDAI and NAFLD.

Subgroups	Quartile 1	Quartile 2	Quartile 3	Quartile 4	*P*_for interaction_
Age					0.397
20–59 years	Ref	0.92 (0.61, 1.41)	0.69 (0.42, 1.12)	0.61 (0.42, 0.88) *	
≥60 years	Ref	0.62 (0.35, 1.09)	0.67 (0.33, 1.33)	0.42 (0.23, 0.78) *	
Sex					0.619
Female	Ref	0.83 (0.46, 1.50)	0.65 (0.42, 1.01)	0.55 (0.32, 0.94) *	
Male	Ref	0.86 (0.52, 1.43)	0.62 (0.38, 1.00)	0.72 (0.51, 1.03)	
Race/ethnicity					0.032
Non-Hispanic White	Ref	0.87 (0.49, 1.56)	0.63 (0.38, 1.06)	0.68 (0.44, 1.07)	
Other races	Ref	0.89 (0.65, 1.21)	0.94 (0.69, 1.27)	0.47 (0.29, 0.75) *	
Education level					0.051
Less than high school	Ref	0.73 (0.40, 1.31)	0.66 (0.26, 1.69)	1.18 (0.56, 2.45)	
High school or above	Ref	0.78 (0.52, 1.18)	0.63 (0.43, 0.90) *	0.52 (0.38, 0.71) *	
Poverty to income ratio					0.908
<2.5	Ref	0.71 (0.47, 1.07)	0.68 (0.42, 1.11)	0.68 (0.48, 0.97) *	
≥2.5	Ref	0.85 (0.49, 1.48)	0.60 (0.40, 0.91) *	0.59 (0.41, 0.84) *	
Smoking status					0.182
No	Ref	0.82 (0.53, 1.27)	0.62 (0.42, 0.92) *	0.52 (0.36, 0.75) *	
Yes	Ref	0.58 (0.28, 1.16)	0.66 (0.32, 1.36)	0.72 (0.29, 1.78)	
Obesity status					0.268
Obesity	Ref	1.08 (0.63, 1.85)	0.81 (0.46, 1.45)	0.75 (0.46, 1.23)	
Non-obesity	Ref	0.47 (0.31, 0.71) *	0.42 (0.26, 0.69) *	0.34 (0.21, 0.57) **	

Adjustments included age, sex, race/ethnicity, total energy intake, BMI, waist circumference, PIR, education, smoking status, alcohol use, exercise, marital status, systolic and diastolic blood pressure, dietary supplement use, and diabetes mellitus status by excluding the corresponding stratified variables. * *p* < 0.05; ** *p* < 0.01.

## Data Availability

The National Health and Nutrition Examination Survey dataset is publicly available at the National Center for Health Statistics of the Center for Disease Control and Prevention (https://wwwn.cdc.gov/nchs/nhanes/default.aspx, accessed on 8 October 2023).

## Supplementary Materials

The following supporting information can be downloaded at: https://www.mdpi.com/article/10.3390/nu16203556/s1, Table S1. Baseline characteristics stratified by Quartile of CDAI (*n* = 6570) in the NHANES.
