# Supplementary material for: Association of the Composite Dietary Antioxidant Index and Consumption Time with NAFLD: The U.S. National Health and Nutrition Examination Survey, 2017–2020
Source: Nutrients. 2024 Oct 20;16(20):3556. doi: 10.3390/nu16203556 (PMC11514589; doi:10.3390/nu16203556)
Supplement: Supplementary file 1 [file nutrients-16-03556-s001.zip › nutrients-3252346-supplementary.pdf]

Supplement Table S1. Baseline characteristics stratified by Quartile of CDAI (n = 6570) in the NHANES

	Quartile 1 (N = 1643)	Quartile 2 (N = 1642)	Quartile 3 (N = 1644)	Quartile 4 (N = 1641)	*P* - value
Age, years	45.93(44.42,47.43)	47.63(46.25,49.00)	46.62(45.18,48.06)	48.06(46.37,49.75)	0.37
Male, %	37.03(33.24,40.82)	38.54(34.71,42.37)	51.17(46.71,55.63)	62.36(59.23,65.48)	< 0.001
Race/ethnicity, %					< 0.001
Non-Hispanic White	59.74(54.72,64.76)	66.58(62.68,70.49)	67.73(62.69,72.77)	64.81(60.94,68.68)	
Non-Hispanic Black	16.26(12.80,19.73)	11.00(8.47,13.53)	9.39(6.92,11.85)	8.59 6.53,10.65)	
Mexican American	7.07(4.61, 9.54)	7.34(5.78, 8.89)	7.00(5.02, 8.99)	8.44(6.22,10.67)	
Other races	16.92(14.10,19.75)	15.08(12.37,17.79)	15.88(12.84,18.92)	18.15(15.55,20.75)	
Smoking status, %					< 0.001
Non - smoker	76.31(72.64,79.99)	85.98(83.23,88.73)	85.47(82.23,88.71)	87.78(85.94,89.63)	
Current smoker	23.69(20.01,27.36)	14.02(11.27,16.77)	14.53(11.29,17.77)	12.22(10.37,14.06)	
Poverty to income ratio, %					< 0.001
< 1.3	22.94(20.41,25.47)	19.68(17.23,22.12)	13.91(11.03,16.79)	14.08(11.80,16.36)	
1.3–3.49	36.77(32.80,40.73)	33.31(28.39,38.23)	30.84(27.01,34.66)	32.65(27.86,37.44)	
≥ 3.5	40.29(36.00,44.58)	47.01(42.29,51.73)	55.25(50.22,60.28)	53.27(48.64,57.90)	
Alcohol status, %					0.07
None	10.30(8.37,12.23)	6.68(5.08, 8.28)	8.26(6.50,10.03)	6.29(4.19, 8.38)	
Low to moderate	64.46(59.99,68.94)	71.36(68.15,74.56)	68.15(63.04,73.25)	72.51(69.12,75.90)	
Heavy	25.24(21.16,29.31)	21.96(18.79,25.14)	23.59(19.32,27.86)	21.20(18.19,24.21)	
Educational level, %					< 0.001
Less than high school	11.20(8.81,13.59)	6.84(5.50, 8.19)	5.61(4.21, 7.01)	7.74(5.77, 9.71)	
High school or equivalent	33.46(29.29,37.63)	29.54(26.69,32.39)	23.28(19.37,27.18)	20.59(18.11,23.08)	
College or more	55.33(50.78,59.89)	63.62(60.54,66.70)	71.12(66.98,75.26)	71.67(68.62,74.71)	
Marital status, %					< 0.001
Never married	24.25(20.61,27.88)	18.81(15.85,21.78)	16.91(14.34,19.48)	16.89(13.40,20.39)	
Widowed/divorced/separated	20.67(17.26,24.08)	19.85(17.43,22.26)	15.82(13.08,18.57)	14.77(12.75,16.78)	
Married/cohabiting	55.08(50.35,59.81)	61.34(57.76,64.92)	67.26(63.86,70.67)	68.34(63.78,72.90)	0.001
Physical activity, %					0.001
Inactive	60.53(57.15,63.91)	50.59(47.89,53.28)	48.74(43.97,53.50)	42.55(38.59,46.51)	
Active	39.47(36.09,42.85)	49.41(46.72,52.11)	51.26(46.50,56.03)	57.45(53.49,61.41)	
Body mass index, kg/m2	30.36(29.80,30.91)	30.03(29.55,30.50)	29.53(28.96,30.10)	29.40(28.70,30.09)	0.05
Total energy intake, kcal/day	1449.01(1408.07,1489.95)	1953.83(1900.06,2007.60)	2317.22(2249.88,2384.56)	2856.83(2764.98,2948.67)	< 0.0001
Waist circumference, cm	101.26(100.09,102.43)	101.10(99.79,102.41)	99.86(98.20,101.53)	100.67(98.78,102.55)	0.52
SBP, mmHg	121.54(119.57,123.51)	120.54(119.56,121.51)	120.14(118.60,121.68)	121.78(120.54,123.03)	0.14
DBP, mmHg	74.82(73.74,75.89)	74.27(73.48,75.07)	73.34(72.37,74.31)	74.06(73.09,75.04)	0.33
CDAI	-3.98(-4.06, -3.91)	-1.66(-1.69, -1.62)	0.53(0.47, 0.58)	5.15(4.91, 5.40)	< 0.001
Dinner CDAI	-2.37(-2.50, -2.24)	-0.83(-0.95, -0.71)	0.58(0.34, 0.82)	2.87(2.45, 3.29)	< 0.001
Lunch CDAI	-2.63(-2.79, -2.48)	-0.97(-1.15, -0.79)	0.24(0.04, 0.44)	3.19(2.92, 3.46)	< 0.001
Breakfast CDAI	-2.13(-2.29, -1.97)	-0.97(-1.16, -0.78)	0.26(-0.03, 0.54)	2.53(2.13, 2.94)	< 0.001
Vitamin A, μ g	278.06(258.82, 297.30)	477.17(449.36, 504.98)	636.03(609.89, 662.18)	1113.63(1054.61,1172.66)	< 0.001
Vitamin C, mg	32.69(30.19, 35.19)	50.87(47.74, 54.01)	81.53(75.68, 87.38)	126.75(119.86,133.63)	< 0.001

Vitamin E, mg	4.76(4.57, 4.95)	7.46(7.12, 7.80)	9.79(9.45,10.13)	15.10(14.30,15.89)	< 0.001
Zinc, mg	6.14(5.95, 6.32)	9.33(9.10, 9.57)	11.79(11.20,12.37)	16.15(15.59,16.71)	< 0.001
Selenium, μ g	70.24(68.34, 72.13)	101.30(98.78,103.82)	123.11(118.91,127.31)	160.25(154.71,165.80)	< 0.001
Carotenoid, μ g	3472.48(3103.25, 3841.70)	6726.75(6225.99, 7227.51)	9934.42(9389.86,10478.97)	17951.00(16103.39,19798.60)	< 0.001
Regular exercise, %	39.47(36.09,42.85)	49.41(46.72,52.11)	51.26(46.50,56.03)	57.45(53.49,61.41)	0.05
Dietary supplements use, %	46.17(42.20,50.14)	54.91(50.99,58.84)	58.60(54.77,62.42)	65.66(63.13,68.20)	0.001

Data are presented as mean (SD) or n (%). CDAI, the composite dietary antioxidant index, DBP, diastolic blood pressure, NAFLD, non-alcoholic fatty liver disease, SBP, systolic blood pressure.
